# Cobalt ferrite nanoparticle intercalated carbon nanotubes for a nanomagnetic ultrasensitive sensor of Cr-VI in water

**DOI:** 10.1063/5.0011911

**Published:** 2020-06

**Authors:** Tassew Mekuria, Syed Khalid, Kathryn Krycka, Markus Bleuel, Himanshu Verma, Haiping Hong, Shashi P. Karna, Dereje Seifu

**Affiliations:** 1 Department of Physics and Engineering Physics, Morgan State University, Baltimore, Maryland 21251, USA; 2 NSLS II, Brookhaven National Laboratory, Upton, New York 11973, USA; 3 NIST Center for Neutron Research, National Institute of Standards and Technology, Gaithersburg, Maryland 20899, USA; 4 Department of Materials Science and Engineering, University of Maryland, College Park, Maryland 20742, USA; 5 Department of Physical Sciences, Nicholls State University, Thibodaux, Louisiana 70301, USA; 6 Department of Material and Metallurgical Engineering, SDSMT, Rapid City, South Dakota 57701, USA; 7 Weapons and Materials Research Directorate, Army Research Lab, APG, Maryland 21005, USA

## Abstract

Nanocomposites of cobalt ferrite (CFO) magnetic nanoparticle intercalated carbon nanotubes (CNTs) are evaluated as a nanomagnetic ultrasensitive sensor for an environmental toxin, hexavalent chromium (Cr-VI). Specifically, the structural and magnetic changes that accompany the infiltration of the CFO/CNT composite by Cr-VI are presented. The extended x-ray absorption fine structure shows that the atomic spacing within the CFO structure changes in the presence of Cr, suggesting that the Cr is incorporated into the nanoparticles. Vibrating sample magnetometry (VSM) reveals that the CFO/CNT composite infiltrated with Cr-VI has a 71% enhancement in saturation magnetization compared with the uninfiltrated CFO/CNTs, while small-angle neutron scattering (SANS) suggests that this magnetic enhancement is not associated with the nanoparticle length-scales, but may arise from longer-ranged clusters. Both VSM and SANS clearly demonstrate that the Cr-doped CFO/CNTs are hysteretic with a net magnetization at remanence that is about 1/3 of saturation, while this hysteresis is absent in the undoped CFO/CNTs. These magnetic differences in either remanence or saturation are promising for the magnetic detection of Cr-VI using CFO/CNT sensors.

## INTRODUCTION

Magnetic nanoparticles of CoFe_2_O_4_ (CFO) intercalated on CNTs form nanocomposites that are excellent candidates for a nanomagnetic ultrasensitive sensor for Cr-VI in water.^1^ Recent studies have indicated that CNT and magnetic nanoparticle nanocomposites exhibit enhanced magnetization when CNTs are intercalated with Co_3_O_4_ and CFO magnetic nanoparticles.^2,3^ The observed enhancement in magnetization is due to proximity induced ferromagnetism in a carbonaceous matter when intercalated with magnetic nanoparticles.^4^ Understanding the magnetic response of CFO/CNT nanocomposites when infiltrated with a small amount of Cr-VI is essential for sensor applications of the nanocomposites, which can be used to detect trace amounts (less than ∼1 ppm) of Cr-VI and other heavy metals.

Low dimensional nonmagnetic materials in proximity to nanomagnetic particles experience induced magnetization resulting in the overall enhancement of the composite system.^3,4^ This phenomenon can be used to design portable and environmentally friendly nanomagnetic sensors to detect environmental contaminants, in particular, heavy metals. Environmental contamination is a significant concern, especially since manufacturing and other human-made activities have significantly contaminated our environment, in some cases, irreversibly. Taking this problem into consideration, the development of ultrasensitive sensors to detect and quantify these contaminants is of utmost importance.^5^ Heavy metals are known to be significant environmental contaminants, and medical research indicates that heavy metal exposure to humans lead to several types of significant health impacts.^6^ In natural waters, Cr-III is most abundant but nontoxic; however, Cr-VI is carcinogenic.^7,8^ Chromium, arsenic, cadmium, mercury, and lead are some of the heavy metals in drinking water that may cause poisoning and cancer and are subject to strict federal limits in drinking water and soil.

Understanding the magnetic response of CFO/CNT composite infiltrated with heavy metals will help the investigation of the health and environmental impacts of the various heavy metals.^9^ In this work, exposing CFO/CNT nanocomposites to Cr-VI by dispersion over the surface of the nanocomposites is used to understand the change in saturation magnetization of the nanocomposites. This crucial information can be used in the detection efforts of Cr-VI and other heavy metals in the environment in much smaller quantities below the standards set by the World Health Organization (WHO) and the US Environmental Protection Agency (EPA).^10^

It is well known that Cr-VI has a negative impact on the environment and human health. Cr-VI is less stable than Cr-III^11^ and can be found in numerous parts of the environment, including air, water, and soil. It is considered extremely dangerous to human health, mainly for workers in the steel and textile industries. Smoking tobacco products increases the chance of exposure to Cr.^11^ Cr-VI can alter genetic materials, thus, causing cancer.^12^ Chromium exposure mainly occurs from drinking contaminated water.^13^ Drinking water sources such as spring water, groundwater, rivers, and lakes could get Cr-ion contaminations through erosion, a natural disaster such as a hurricane, and wastewater from textile, coloring, metal, and mining industries.^14^ Cr is the only antiferromagnetic element in the periodic table, and it is known to display different magnetic properties depending on temperature and pressure.^15^

In previous work, vibrating sample magnetometry (VSM) characterization of CFO/CNT composite infiltrated with Cr-VI exhibited saturation magnetization enhancement compared to pristine CFO/CNT nanocomposites.^16^ Understanding the magnetic response of CFO/CNT nanocomposites upon infiltration with a small amount of Cr-VI is essential for developing technologies to detect environmental contamination with Cr-VI and other heavy metals.

## EXPERIMENT

CFO/CNT nanocomposite samples were prepared from a two-to-one ratio of CFO nanoparticles of the mean diameter of 42 nm and 10 *μ*m–50 *μ*m length CNTs, described elsewhere.^1^ Then, 0.2 g of K_2_Cr_2_O_7_ (Sigma-Aldrich), which contains 0.077 *μ*g of chromium, was mixed in 65 ml of deionized water by magnetic stirring for 1 h, and 90 *μ*l of the solution was transferred to the CFO/CNT composite to disperse over the surface of CFO/CNT nanoparticles. CFO/CNT and CFO/CNT-Cr (resulting in a 0.02 ratio of Cr/CoFe_2_O_4_ and 0.31 ratio of Cr/Co) samples were prepared for Small-Angle Neutron Scattering (SANS) and Extended X-ray Absorption Fine Structure (EXAFS) characterization.

**FIG. 1.**
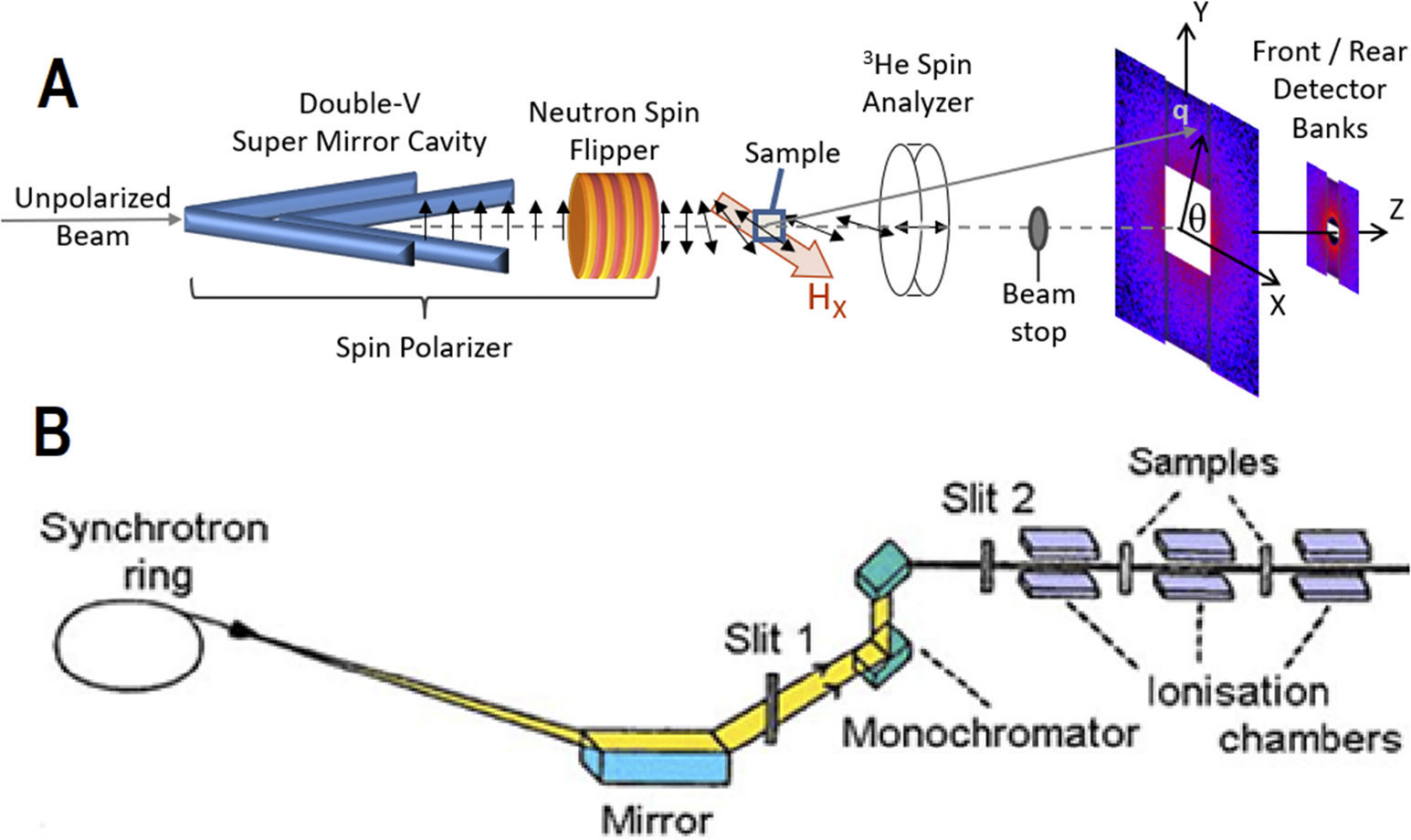
(a) Polarization analyzed small-angle neutron scattering on the vSANS instrument involving the neutron spin polarization components of a super-mirror, radiofrequency spin flipper, and a ^3^He spin analyzer. The neutron spins follow the applied magnetic field as shown unless they encounter abrupt magnetic changes such as within the sample. (b) EXAFS instrumental anatomy.

SANS samples were powder-packed into thin-walled Al sample cans and measured on the vSANS beamline at the NIST Center for Neutron Research at a wavelength of 0.55 nm and a full-width at the half-maximum wavelength spread of 12%. Data were simultaneously collected in two-dimensional detector banks located at 4.9 m and 17.4 m from the sample position, covering a reciprocal space range of 0.003 Å^−1^ to 0.12 Å^−1^ (probing structures on the order of 5 nm–200 nm). The samples were placed in between the poles of a horizontal electromagnet yielding spatially uniform fields between 0.007 T (remanence) to 1.5 T, as shown in Fig. 1(a).

Neutron scattering probes the ensemble averages of both structural (i.e., nuclear) and magnetic morphologies. However, in cases where the structural scattering dominates over the magnetic scattering, neutron spin polarization analysis can be used to effectively separate these two components^17,18^ and highlight the directional dependence of the magnetic scattering. Here, ↑ and ↓ represent neutrons whose spins precess parallel and anti-parallel, respectively, to an applied external magnetic field. Neutron spin polarization was selected prior to the interaction with the sample via an in-beam FeSi super-mirror polarizer cavity and a radio frequency spin flipper, while the relative amounts of spin polarization after scattering from the sample were measured with a ^3^He neutron spin filter combined with an *in situ* NMR flipper.^19^ The efficiency of each polarizing element, though high, is fully corrected, as described in Ref. 20. This results in a total of four spin cross sections: ↑↑, ↑↓, ↓↓, and ↓↑, where the arrows refer to neutron spins before and after the sample, respectively. In short,^17^ ↑↑ + ↓↓ taken along the vertical direction (⊥H and ‖Y in Fig. 1) measure structural scattering plus scattering from moments aligned parallel to H, while ↑↓ + ↓↑ taken along the vertical direction measures only scattering from magnetic moments perpendicular to H. The latter is multiplied by a factor of two to account for the fact that this procedure measures only half the moments not aligned along H (i.e., moments ‖Z, but not moments ‖Y). Additionally, ↓↓−↑↑ also taken along the vertical direction [⊥H and ‖Y in Fig. 1(a)] is a measure of structural-magnetic cross-term involving moments aligned ‖H.^17,21^ In practice, sector cuts of ±15^○^ were taken about the vertical (Y) direction, where the component of M‖H is proportional to $\sin^{2}(\text{θ})$ and θ is the angle from the positive x-axis shown in Fig. 1(a).

EXAFS is an advanced and widely used method for studying atoms and their local environments. EXAFS uses the x-ray photoelectric effect and the wave nature of the electron to determine local structures around the atom in solid and nanomaterials.^22^ EXAFS has become more applicable to investigate the electrochemical and magnetic nature of magnetic nanoparticles.^23^ The EXAFS area from the entire range is characterized by a function χ, defined in terms of the absorption coefficient, as shown in the following equation:

(1) $$\text{χ}(E)=\frac{\text{μ}(E)-\text{μ}0(E)}{\text{μ}o(E)},$$

where μ(E) is a function of energy or excess energy, and ${\text{μ}}_{\text{o}}(\text{E})$ is the initial x-ray absorption energy at the edge. EXAFS is a technique used to measure the molecular parameters of materials^24^ and to study the local structures and movements of atoms during chemical reactions. Here, a high penetration depth by fine-tuning the EXAFS energy range was achieved.^25^

The QAS 7BM beamline at the National Synchrotron Light Source II (NSLS-II) of Brookhaven National Laboratory as shown in the schematic diagram in Fig. 1(b) was used to investigate the magnetic characteristics of the CFO/CNTs infiltrated with Cr-VI. Our samples were prepared based on a requirement set by the BNL for EXAFS measurements.^26^

## RESULTS AND DISCUSSIONS

**FIG. 2.**
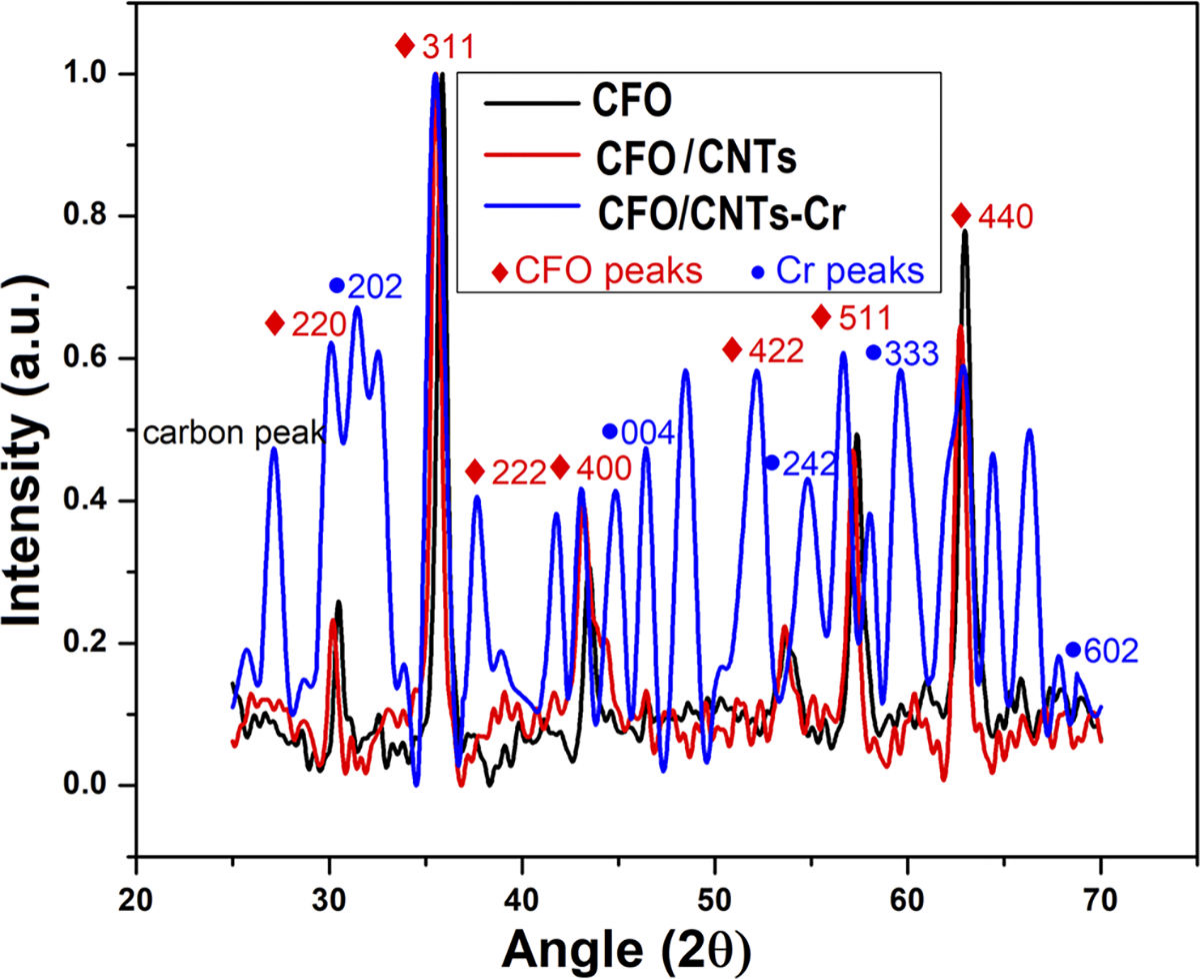
XRD of CFO (black), CFO/CNTs (red), and CFO/CNT-Cr (blue). CFO peaks are in red, and Cr peaks are in blue diamond scatter.

**FIG. 3.**
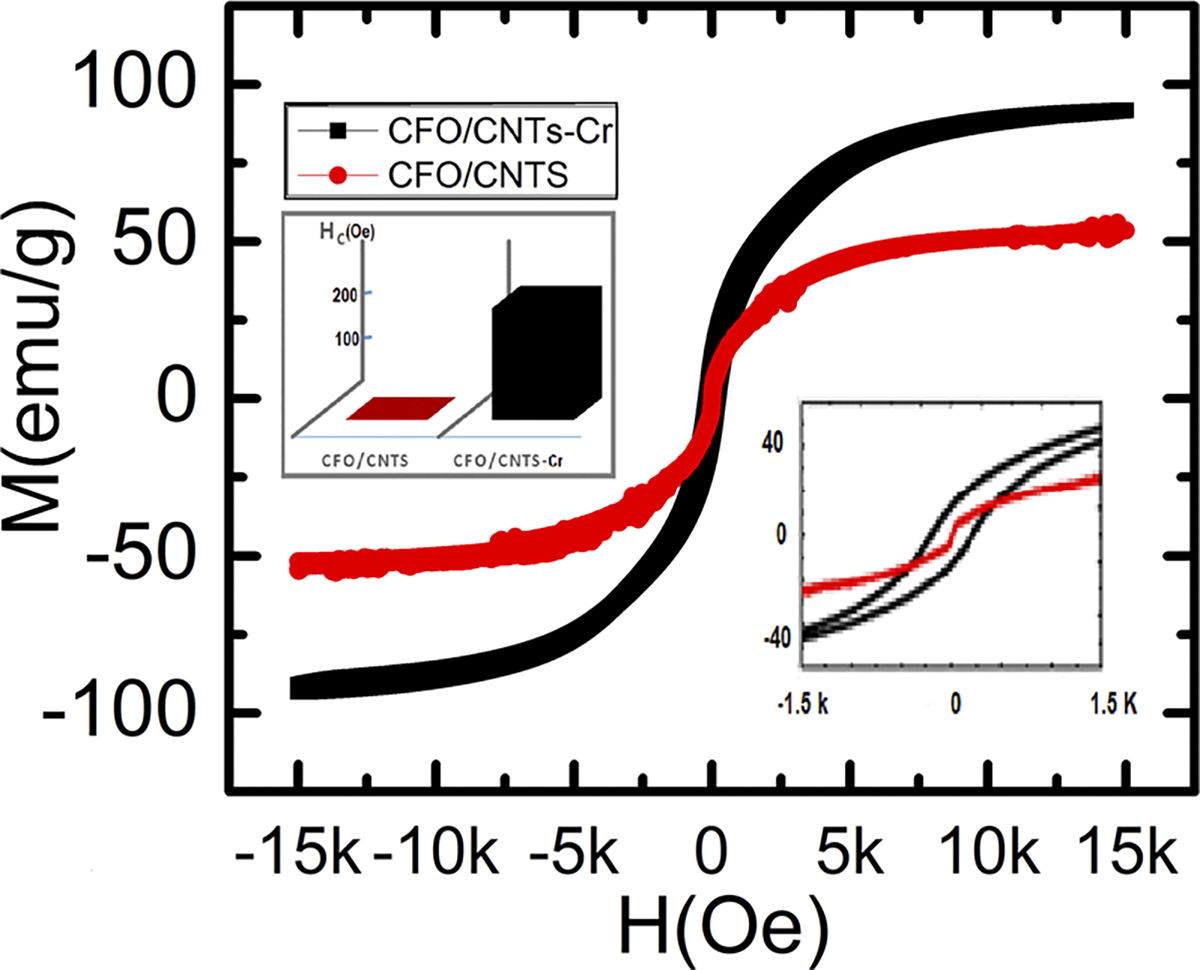
Hysteresis loop of CFO/CNT (red) and CFO/CNT-K_2_Cr_2_O_7_ (black), magnetization M in emu/g of CFO, and applied field in Oesterd (Oe).

Neutron scattering of magnetic materials, such as CFO, provides us essential information on the magnetic and nuclear cross sections.^27^ XRD of CFO, CFO/CNTs, and CFO/CNTs-Cr is shown in Fig. 2 with the corresponding CFO and Cr peaks. In our previous research work, we reported that the magnetic characteristics, especially saturation magnetization of CFO/CNTs infiltrated by a minute amount of Cr-VI, exhibited 71% enhancement compared to the pristine CFO/CNT nanocomposites as shown in Fig. 3.^3^

**FIG. 4.**
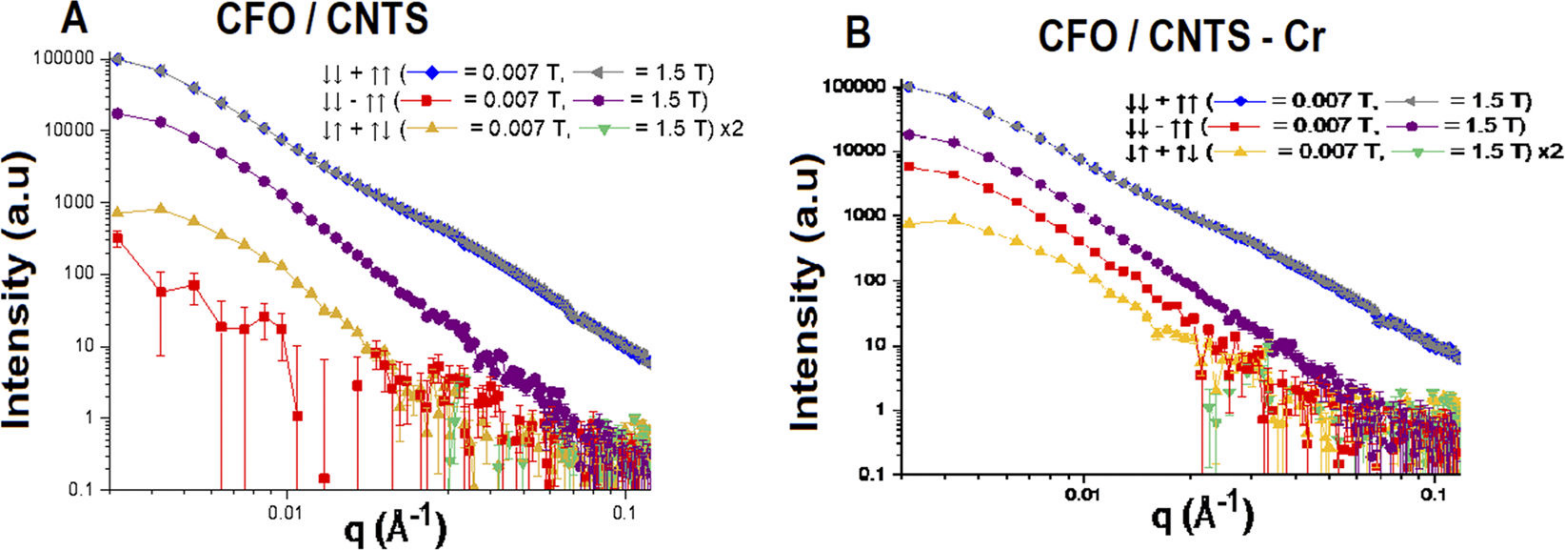
Polarization-analyzed SANS of (a) CFO/CNTs and (b) CFO/CNT-Cr. The blue and gray curves are dominated by structural scattering and do not change significantly with an applied magnetic field. Red and purple curves are proportional to the magnetic scattering from moments aligned with the applied field at 0.007 T and 1.5 T, respectively. The gold curves arise from a scattering of magnetic moments not aligned with the magnetic field at 0.007 T. In contrast, the green curves show that almost no scattering is present from moments not aligned with the applied field at 1.5 T. Error bars shown here and elsewhere represent one standard deviation.

**FIG. 5.**
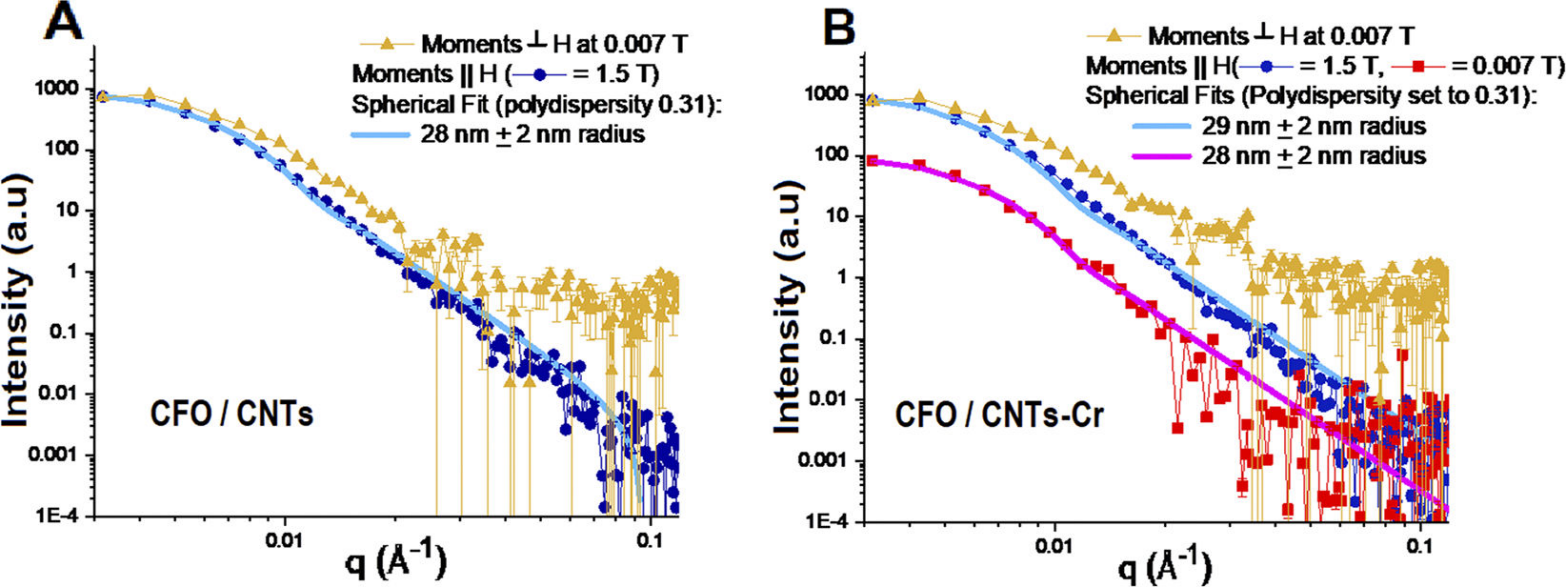
Magnetic-only SANS of (a) CFO/CNTs and (b) CFO/CNT-Cr. The primary difference between the two samples is that a measurable scattering curve exists for the CNT–CFO–Cr sample at 0.007 T [red curve in (b)], which is absent in the CNT–CFO sample.

The polarized SANS data from CFO/CNTs and CFO/CNT-Cr samples are shown in Fig. 4. The precise scaling between the two samples is not known due to variations in powder packing. Thus, a uniform correction was applied to each sample to bring the structural scattering at 0.007 T at the lowest Q-measurement of 0.003 Å^−1^ to even 100 000 counts (for the ease of direct comparison). In both the CFO/CNTs and CFO/CNT-Cr samples, we see that there is no significant difference between ↓↓ + ↑↑ scattering at 0.007 T and 1.5 T, indicating that the sample scattering is dominated by structural contributions rather than magnetic scattering contributions, blue and gray curves of Fig. 4. It is also evident in both samples that the magnetic-only spin-flip scattering (↑↓ + ↓↑), which arises from the component of magnetic moments not aligned with the applied external field, is significant at 0.007 T (though a factor of about 100 lower than the structural scattering), but not at 1.5 T, yellow and green curves of Fig. 4. This implies that at 1.5 T, the magnetic moments are fully aligned with the applied field. The (↓↓ − ↑↑) scattering is similar for the two samples at 1.5 T, yet differs markedly at 0.007 T. The fact that this signal, proportional to moments ‖H, is negligible in the CFO/CNTs at 0.007 T means that almost no net moment ‖H persists at remanence. Yet, its presence in the CFO/CNT-Cr sample indicates that a net magnetization persists at remanence. This is in agreement with the magnetometry results in Fig. 3. Moreover, in the CFO/CNT-Cr, the (↓↓ − ↑↑) signal is 3.1 ± 0.1 times larger at low Q (up to 0.01 Å^−1^) at 1.5 T than at remanence, which is also in general agreement with magnetometry. However, the ratio of (↓↓−↑↑)/(↓↓ + ↑↑) at 1.5 T of Fig. 5 is proportional to the magnetism ‖H/structural scattering, and it is very similar in the samples with peak ratios of 0.20 at 0.005 Å^−1^. This suggests that the saturation magnetization is almost the same for the CFO/CNTs and CFO/CNT-Cr samples within the reciprocal space probed by SANS. Instead, the large saturation magnetization enhancement measured by VSM with the addition of Cr-IV might be correlated with very large magnetic clusters.

The magnetic scattering can be further refined into components arising from magnetism ⊥H and magnetism ‖H, see Fig. 5. Assuming that the magnetism ‖H is correlated with the structural scattering, the magnetic scattering ‖H can be estimated as ${\frac{1}{4}\left(↓,↓,-,↑,↑\right)}^{2}/\left(↓↓+↑↑\right)$,^17^ as shown in Fig. 5 (blue and red curves). Again, the magnetic scattering at 1.5 T is similar in magnitude and shape for both the CFO/CNTs and CFO/CNT-Cr samples. Using Sasview,^21^ this can be fit to magnetic nanoparticle spheres of radius (28 ± 2) nm and (29 ± 2) nm, respectively, with a 0.31 full-width half-maximum polydispersity. The residual magnetic moments at 0.007 T for the CFO/CNT-Cr sample also fit a similar model of (28 ± 2) nm magnetic spheres. Here, the difference between the magnetic scattering at 0.007 T and 1.5 T is a function of the magnetic moments squared. While the magnetic model predicts magnetic particles slightly larger than the expected 42 nm in diameter, we note that the modeling is highly dependent upon the polydispersity of the nanoparticles such that more polydispersity would yield a lower mean diameter. The models do reveal a consistent magnetic size for the moments aligned ||H across the samples and at different applied magnetic fields. Finally, the moments perpendicular to H (gold curves of Fig. 5) have a much higher spin-flip background, likely due to the incoherent scattering associated with hydrogen (which is removed from the processing of the magnetism ‖H scattering during the ↓↓ − ↑↑ subtraction step). However, the scattering from these moments perpendicular to H at 0.007 T is similar in magnitude to that from the moments ‖H at 1.5 T, showing rough conservation of moments and a tendency for the moments to stay aligned within the individual nanoparticles even as remanence.

**FIG. 6.**
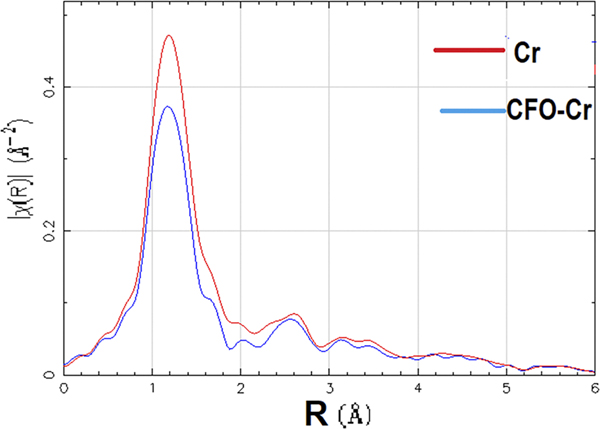
EXAFS measurements showing the difference in the Fourier transform of CFO with Cr (blue) and Cr (red).

**FIG. 7.**
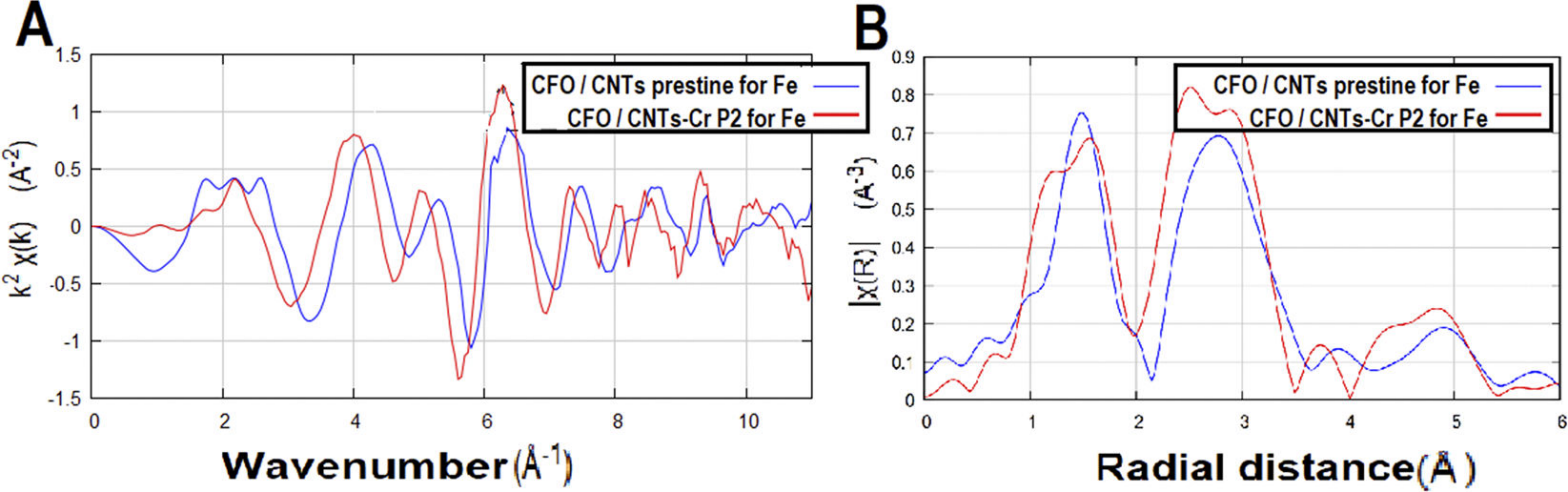
EXAFS measurements showing (a) the wave vector and (b) radial distance comparison of the pristine CFO/CNTs for Fe and CFO/CNTs with Cr for the energy of Fe.

The EXAFS characterization of CFO/CNTs infiltrated with Cr-VI turns out to be radically different from the one observed for pristine CFO/CNTs. The fingerprinting analysis of the data is shown in Figs. 6 and 7. The data range was from −200 eV to 525 eV, relative to the K-edge of iron, and the k range was from 3 to 11 using the Hanning window, and the k2 weighting was used.^28^ The difference can be explained either in terms of distinct intercalation sites between both samples or in terms of different interactions between O, Fe, Co, and Cr.

As a general principle, the x rays of specific energy are absorbed, removing a core electron of the K-shell. In our sample, the core electron was from the S-shell of iron. The x-ray energy to knock out this electron is about 7112 eV. As the x-ray energy keeps increasing, this electron wave keeps taking the excess energy and interacts with surrounding atoms and scatters back to the absorbing atom. The constructive and destructive interference at the absorber gives rise to the EXAFS pattern. Mathematical massaging of this pattern infers information about the atomic distances and coordination numbers.

The CFO has a spinel crystal structure. The O and Fe atoms are at tetrahedral and octahedral sites. The Fourier transform of the CFO/CNT pristine original for Fe indicates that about 40% occupy the tetrahedral site and 60% occupy the octahedral site.^29^ The structure of CFO, using EXAFS, has been reported for two Fe–O, Fe–Fe, Fe–Co, and Co–O distances with their coordination numbers for this spinel crystal, calcined at 800 ^○^C.^30^ The reported distances are Fe–O—1.88 Å, Fe–O—1.99 Å, Fe–Fe—2.96 Å, and Fe–Co—3.47 Å, and their observed coordination numbers are 1.87, 0.86, 3.15, and 2.11, respectively.

Fundamental differences were observed in the Fe data of the two datasets, CFO/CNTs not infiltrated by Cr-VI, and pristine CFO/CNTs. The radial distribution plot of CFO/CNTs in Fig. 7, without phase correction, shows the first peak at 1.49 Å; this is the peak corresponding to the Fe–O distance in the sample as deposited on carbon nanotubes. The second peak at 2.78 Å is likely the Fe–Fe distance. These peaks split when Cr-VI is added. The first peak splits into two, at 1.22 Å and 1.56 Å. Either the Fe–O distance has been reduced, and an O is at 1.22 Å and Cr at 1.56 Å, or it has been expanded, and a Cr atom is at 1.22 Å and O at 1.56 Å. In the CFO/CNT sample, the second peak was at 2.78 Å. With the addition of Cr-VI, the distance splits to 2.50 Å and 2.84 Å, which is one lower and one higher distance from the original second shell of iron in the CFO/CNT sample. Either, the lower one is a shrunk Fe atom and the higher one a Cr atom, or it could be vice versa. The peak height differences of the two major peaks of the radial distribution, when adding K_2_Cr_2_O_7_, also indicate a redistribution of the octahedral and tetrahedral sites—apparently about 90% tetrahedral and 10% octahedral.^29^

## CONCLUSIONS

The infiltration of Cr-VI into cobalt ferrite magnetic nanoparticles intercalated on carbon nanotubes (CFO/CNTs) reveal interesting magnetic differences at both saturation and remanence, suggesting that CFO/CNTS composites could be harnessed to detect environmental contamination by Cr. EXAFS show structural changes between CFO/CNTs with and without Cr-IV infiltration, indicating that the Cr has been incorporated into the CFO structure. At magnetic saturation (1.5 T), VSM showed a significant 71% magnetic enhancement in the CFO/CNT composite containing Cr-IV, while the SANS showed that this magnetic difference was not correlated with the local magnetic enhancement of the cobalt ferrite nanoparticles. Thus, the VSM may be sensitive to a magnetic enhancement correlated with much larger structures. At remanence (up to 0.007 T), both the VSM and SANS revealed that the CFO/CNT-Cr nanoparticles were hysteretic with a residual magnetization about 1/3 that of saturation, while the CFO/CNT composites were not hysteretic and did not contain a net remanent magnetization. Thus, the CFO/CNT architecture offers two ways to potentially detect the environmental Cr contamination: through an increase in the long-range net magnetization at saturation or by imparting a residual magnetization within the CFO nanoparticles after exposure to a magnetic field.

## Data Availability

The data that support the findings of this study are available from the corresponding author upon reasonable request.
